# Pilates exercises for managing premenstrual syndrome among adolescent girls

**DOI:** 10.6026/973206300210127

**Published:** 2025-02-28

**Authors:** Vaghela Payalben Tejmalji, Mahalakshmi B., Siva Subramanian N.

**Affiliations:** 1 Department of Obstetrics and Gynecological Nursing, Nootan college of Nursing, Sankalchand Patel University, Visnagar, Gujarat - 384315, India; 2 Department of Pediatric Nursing, Nootan College of Nursing, Sankalchand Patel University, Visnagar, Gujarat - 384315, India; 3 Department of Psychiatric Nursing, Nootan College of Nursing, Sankalchand Patel University, Visnagar, Gujarat - 384315, India

**Keywords:** Premenstrual syndrome, pilates, adolescent girls, non-pharmacological interventions, holistic health

## Abstract

Premenstrual Syndrome (PMS) is a common condition among adolescent girls, characterized by physical, emotional and behavioural symptoms that significantly impact their quality of life. Therefore, it is of interest to evaluate the impact of pilates exercises on alleviating PMS symptoms among adolescent girls, providing evidence for its use as a holistic intervention. A quasi-experimental pretest-posttest design was employed; involving 231 adolescent girls aged 13-17 years from selected schools in North Gujarat. Participants underwent a six-week pilates exercise program, conducted five times weekly. Data were collected using a demographic questionnaire and the Modified PMS Scale to assess pre- and post-intervention symptoms the intervention led to a significant reduction in PMS severity, with mild PMS cases increasing from 50 to 164 and severe cases decreasing from 22 to 10 post-intervention. Paired t-tests revealed a highly significant mean difference in PMS scores (T = 13.122, p < 0.001).

## Background:

Premenstrual syndrome (PMS) is a common condition affecting adolescent girls, characterized by a range of physical, emotional and behavioral symptoms occurring during the luteal phase of the menstrual cycle [1]. These symptoms, including abdominal cramps, mood swings, irritability and fatigue, can significantly impact the quality of life, academic performance and social interactions of young girls [2]. Globally, the prevalence of PMS ranges between 30% and 50%, with nearly 20% of cases classified as severe. Despite its widespread impact, PMS often remains underdiagnosed and inadequately managed, particularly among adolescents [3]. While conventional treatments such as medications and dietary adjustments offer relief, there is a growing interest in holistic, non-pharmacological approaches that empower individuals to manage their symptoms effectively [4]. pilates exercises, originally developed by Joseph Pilates, are a form of low-impact physical activity that focuses on core strength, flexibility, posture and controlled breathing. By enhancing physical and mental well-being, pilates has emerged as a promising intervention for alleviating various health conditions, including stress and musculoskeletal discomfort [5]. In the context of PMS, pilates exercises may offer unique benefits by addressing both the physical discomforts and the emotional challenges associated with the condition. Improved circulation, enhanced muscle relaxation and reduced stress levels are potential mechanisms through which pilates could alleviate PMS symptoms. Despite its potential, limited research has explored the effectiveness of pilates exercises specifically for adolescent girls with PMS. Therefore, it is of interest to evaluate the impact of pilates exercises on PMS among adolescent girls, providing evidence for its use as a viable, non-pharmacological intervention. By targeting this vulnerable demographic, the research seeks to contribute to the growing body of knowledge on holistic PMS management and inform future health promotion strategies tailored to adolescents.

## Methodology:

## Research design and setting:

A quasi-experimental, pretest-posttest design was conducted [5-6] in selected schools in North Gujarat, providing a structured and accessible environment for the intervention. Population and Sampling the study targeted adolescent girls aged 13-17 years experiencing PMS. A sample of 231 participants was selected through stratified random sampling and assigned to a single intervention group.

## Intervention:

Participants engaged in a six-week pilates exercise program, consisting of warm-up, core exercises and cool-down sessions conducted five times per week by a certified instructor. Data were collected using a demographic questionnaire and the Modified PMS Scale to assess symptoms. Ethical approval was obtained, informed consent was secured and participant confidentiality was maintained throughout the study.

## Data analysis:

Descriptive statistics summarized data, while paired t-tests assessed changes in PMS symptoms pre - and post-intervention. The primary outcome was a reduction in PMS symptoms, evidenced by lower scores on the Modified PMS Scale.

## Results:

Table 1 show that the majority being 15 years old (31.6%). Most participants were Hindu (46.32) and 53.68% followed a vegetarian diet. The most common weight range was 41-45 kg (27.27%) and 39.83% were firstborn children. Nearly half (48.48%) reported engaging in physical exercise and 50.65% had 8-10 hours of sleep daily. Menarche was attained after 12 years for 58.87% and most reported menstrual flow lasting 5-7 days (40.26%) with less than 4 pads used per day (45.02%). A family history of PMS was present in 31.6% and the majority experienced PMS for 4-6 days (51.52%). Figure 1 (see PDF) show Mild PMS cases increased significantly from 50 participants in the pretest to 164 in the posttest. Conversely, moderate PMS cases decreased from 148 to 52 and severe PMS cases dropped from 22 to 10. Very severe cases were reduced from 11 participants to 5. This indicates that Pilate's exercises had a substantial positive impact on PMS severity levels. Table 2 revealed significant associations between PMS severity and certain demographic variables, including age (χ^2^ = 20.657, p = 0.014), frequency of menstrual cycle (χ^2^ = 17.854, p = 0.037), days of menstrual flow (χ^2^ = 24.025, p = 0.001), family history of PMS (χ^2^ = 13.241, p = 0.004) and days with PMS (χ^2^ = 33.345, p = 0.001). Other variables, such as religion, diet, weight, birth order, type of beverage, physical exercise, hours of sleep, menarche and pads changed per day, showed no significant associations with PMS severity (p > 0.05). These findings indicate that menstrual-related factors and family history play a critical role in influencing PMS severity. The paired t-test showed a statistically significant mean difference of 20.164 between pretest and posttest scores (T = 13.122, P < 0.001). This confirms the effectiveness of pilates exercises in reducing PMS symptoms, with a highly significant reduction in severity after the intervention.

## Discussion:

Premenstrual syndrome (PMS) significantly affects the quality of life for many adolescent girls, yet effective and accessible non-pharmacological treatments remain limited. This study demonstrated that a six-week Pilates exercise intervention significantly reduced PMS symptoms in adolescent girls. Çitil and Kaya (2021) demonstrated that Pilates exercises were effective in reducing premenstrual syndrome symptoms in their quasi-experimental study [7]. Similarly, Sanchez *et al.* (2023) highlighted in their narrative review that exercise, in general, was effective in alleviating premenstrual syndrome symptoms [8]. The current studies results align with research by Çitil *et al.* [7], which showed that Pilate's exercises significantly reduced PMS symptoms in a quasi-experimental study involving university students. Similarly, Balmumcu *et al.* [9] demonstrated that an intervention combining Pilates and a WhatsApp-based support program effectively reduced PMS severity, emphasizing the mental and physical benefits of pilates as part of a holistic intervention. The reduction in PMS symptoms observed in the intervention group could be attributed to improved circulation, muscle relaxation and reduced cortisol levels, as noted in previous studies. For instance, Omidali *et al.* [10] reported that pilates not only alleviated mood and physical symptoms of PMS but also enhanced participants overall functionality. While the current study focused on Pilates, other forms of exercise, including aerobic and yoga practices, have demonstrated similar benefits for PMS management. Vaghela *et al.* [11] found that yoga outperformed aerobic exercises in reducing PMS symptoms, though both forms of exercise were beneficial. These findings highlight Pilates as a complementary intervention alongside other physical activities. The study by Song *et al.* [12] emphasized the role of Pilates in improving sleep quality, reducing stress and enhancing overall physical function, which are critical factors in mitigating PMS symptoms. Additionally, exercise such as yoga has been shown to decrease anxiety and depressive symptoms associated with PMS, as observed by Ghaffarilaleh *et al.* [13].

The current study identified significant associations between PMS severity and factors such as age, family history and menstrual-related variables. These findings align with prior work by Saglam *et al.* [14], who highlighted the importance of demographic and lifestyle factors in influencing PMS symptoms. The current study found a significant association between age and PMS severity (p=0.014). Similar findings were reported by Timonen *et al.* [15], who noted that younger women tended to experience more emotional and behavioral symptoms compared to physical discomforts in older age groups. However, other research, such as Lu *et al.* [16], suggested that PMS severity might peak during late adolescence due to hormonal fluctuations and the transition from irregular to more stable cycles. Our results revealed that the frequency of the menstrual cycle significantly influenced PMS severity (p=0.037). This aligns with findings from [15] Harlow *et al.* [17], who emphasized that shorter menstrual cycles often lead to increased hormonal fluctuations, contributing to more severe symptoms. Additionally, the study by Nazir *et al.* [18] highlighted that Pilates exercises helped regulate the hormonal shifts during the luteal phase, reducing both physical and psychological symptoms. A strong association was observed between the duration of menstrual flow and PMS severity (p=0.001). This finding is consistent with Dawood [19], who noted that longer menstrual flows correlated with increased premenstrual discomfort, likely due to heightened prostaglandin release. Moreover, the study by Jensen *et al.* [20] highlighted that regular exercise, including Pilates, reduced abdominal cramping and bloating, symptoms that are often exacerbated by extended menstrual flow durations. Our findings indicate that a family history of PMS significantly increases symptom severity (p=0.004). This observation is supported by Safarzadeh *et al.* [21], who found that hereditary factors and lifestyle habits often predispose individuals to more pronounced PMS symptoms.

## Figures and Tables

**Table 1:** Demographic characteristics of the pilates exercise group

**Demographic Variable**	**Categories**	**Frequency (n)**	**Percentage (%)**
Age	13 years	53	22.94
	14 years	55	23.81
	15 years	73	31.6
	Above 15 years	50	21.65
Religion	Hindu	107	46.32
	Muslim	55	23.81
	Christian	30	12.99
	Others	39	16.88
Diet	Vegetarian	124	53.68
	Non-Vegetarian	107	46.32
Weight (kg)	Less than 31 kg	17	7.36
	31-35 kg	46	19.91
	36-40 kg	51	22.08
	41-45 kg	63	27.27
	46-50 kg	48	20.78
	Above 50 kg	6	2.6
Birth Order	First Child	92	39.83
	Second Child	67	29
	Third Child and Above	72	31.17
Beverage Taken Often	Coffee	41	17.75
	Tea	46	19.91
	Milk	59	25.54
	Fruit Juice	40	17.32
	None	45	19.48
Physical Exercise	Yes	112	48.48
	No	119	51.52
Hours of Sleep per Day	Less than 5 hours	8	3.46
	5-7 hours	106	45.89
	8-10 hours	117	50.65
	More than 10 hours	0	0
Age at Menarche	Less than 12 years	95	41.13
	More than 12 years	136	58.87
Frequency of Menstrual Cycle	26-28 days	59	25.54
	29-31 days	61	26.41
	32-34 days	57	24.68
	Above 35 days	54	23.37
Days of Menstrual Flow	2-4 days	82	35.5
	5-7 days	93	40.26
	8-10 days	56	24.24
Pads Changed per Day	Less than 4 pads	104	45.02
	4-5 pads	82	35.5
	More than 5 pads	45	19.48
Family History of PMS	Present	73	31.6
	Absent	158	68.4
Days with PMS	1-3 days	68	29.44
	4-6 days	119	51.52
	7-10 days	42	18.18
	More than 10 days	2	0.86

**Table 2:** Association between pretest PMS scores and socio-demographic variables

**Demographic Variable**	**Chi-Square Value**	**DF**	**P-Value**	**Result**
Age	20.657	9	0.014	Significant
Religion	8.981	9	0.439	Not Significant
Diet	2.679	3	0.444	Not Significant
Weight (kg)	9.018	15	0.877	Not Significant
Birth Order	7.908	6	0.245	Not Significant
Type of Beverage	9.141	12	0.691	Not Significant
Physical Exercise	8.4	3	0.038	Not Significant
Hours of Sleep Per Day	1.05	6	0.984	Not Significant
Menarche	1.887	3	0.596	Not Significant
Frequency of Menstrual Cycle	17.854	9	0.037	Significant
Days of Menstrual Flow	24.025	6	0.001	Significant
Pads Changed per Day	8.58	6	0.199	Not Significant
Family History of PMS	13.241	3	0.004	Significant
Days with PMS	33.345	9	0.001	Significant
